# Dunglison’s New Pronouncing Dictionary

**Published:** 1893-10

**Authors:** 


DUNGLISON'S NEW PRONOUNCING MEDICAL DICTIONARY.—A new edition of Dunglison's Medical Dictionary is announced as in press for early publication. It has been thoroughly revised and greatly enlarged, and will contain about forty-four thousand new medical words and phrases. Pronunciation has been introduced into the new edition by means of a simple phonetic spelling. This work has always been noted for the fullness of its definitions, ample explanation being its distinguishing characteristic. In the new edition much encyclopedic information, difficult of access elsewhere, will be found conveniently at hand. Especial attention has been devoted to matters of practical value A review will appear in an early issue.

